# Factors Influencing Antiretroviral Adherence and Virological Outcomes in People Living with HIV in the Highlands of Papua New Guinea

**DOI:** 10.1371/journal.pone.0134918

**Published:** 2015-08-05

**Authors:** Janet Gare, Angela Kelly-Hanku, Claire E. Ryan, Matthew David, Petronia Kaima, Ulato Imara, Namarola Lote, Suzanne M. Crowe, Anna C. Hearps

**Affiliations:** 1 Sexual and Reproductive Health Unit, Papua New Guinea Institute of Medical Research, Goroka, Eastern Highlands Province, Papua New Guinea; 2 Centre for Biomedical Research, Burnet Institute, Melbourne, Victoria, Australia; 3 Central Clinical School, Monash University, Melbourne, Victoria, Australia; 4 School of Public Health and Community Medicine, University of New South Wales, Sydney, New South Wales, Australia; 5 HIV/STI Highlands Region, National Department of Health, Mt Hagen, Western Highlands Province, Papua New Guinea; 6 Michael Alpers Clinic, Goroka General Hospital, Goroka, Eastern Highlands Province, Papua New Guinea; Alberta Provincial Laboratory for Public Health/ University of Alberta, CANADA

## Abstract

Adherence to antiretroviral therapy (ART) is paramount for virological suppression and positive treatment outcomes. ART has been rapidly scaled up in Papua New Guinea (PNG) in recent years, however clinical monitoring of HIV+ individuals on ART is limited. A cross-sectional study was conducted at two major sexual health clinics in high HIV prevalence provinces in the Highlands Region of PNG to assess ART adherence, factors affecting adherence and the relationship between ART adherence and virological outcomes. Ninety-five HIV+ individuals were recruited and administered a questionnaire to gather demographic and ART adherence information whilst clinical data and pill counts were extracted from patient charts and blood was collected for viral load testing. Bivariate analysis was performed to identify independent predictors of ART adherence. Fourteen percent (n = 12) of participants showed evidence of virological failure. Although the majority of participants self-reported excellent ART adherence in the last seven days (78.9%, 75/91), pill count measurements indicated only 40% (34/84) with >95% adherence in the last month. Taking other medications while on ART (p = 0.01) and taking ART for ≥1 year (p = 0.037) were positively associated with adherence by self-report and pill count, respectively. Participants who had never heard of drug resistance were more likely to show virological failure (p = 0.033). Misconception on routes of HIV transmission still persists in the studied population. These findings indicate that non-adherence to ART is high in this region of PNG and continued education and strategies to improve adherence are required to ensure the efficacy of ART and prevent HIV drug resistance.

## Introduction

Located in the South Pacific and sharing the eastern side of the island of New Guinea with Indonesia (West Papua) is the Independent state of Papua New Guinea (PNG) with the landmass area of 452,860 square kilometres. The country is inhabited by more than 7 million people speaking over 800 different languages and has the greatest burden of HIV in the Pacific region with an estimated 31, 945 people living with HIV (PLHIV) in 2013.[1]Although estimates and projections made in 2005–6 predicted an epidemic similar to sub-Saharan Africa, improvements in HIV testing and reporting systems has revised the national HIV prevalence downwards to less than 1% with some estimates at 0.65% in adults aged 15–49 years old.[1–3] While relatively low in the general population, reports from certain geographic locations and most at risk populations indicate higher HIV prevalence of 2.3%-19%. [4–7] As of 2012, the HIV epidemic in PNG was described as a concentrated epidemic and has prompted the National Department of Health to refocus its priority activities in HIV research and intervention. However, others argue that the epidemiological evidence suggests a mixed epidemic, rather than concentrated or generalised epidemic. [8,9]

Antiretroviral therapy (ART) was first introduced in PNG in 2004 through a pilot project at the Heduru Clinic in the capital city, Port Moresby; the subsequent national roll-out began in 2006. At present, there are more than 90 registered sites providing ART throughout PNG with an estimated treatment coverage of 74% (n = 11,764) of those in need of ART [10] as defined by having a CD4 T-cell count of ≤350/mm^3^ based on the 2010 WHO treatment guidelines for resource limited settings. The majority of PLHIV on ART in PNG receive first line therapy, which consists of a combination of zidovudine or tenofovir, plus lamivudine and nevirapine or efavirenz. Stavudine was initially prescribed in first line regimens but due to its high degree of toxicity, was recently replaced with tenofovir. The use of protease inhibitors is rare. Monitoring of treatment outcomes is based almost exclusively on clinical symptoms and although CD4 testing is available in some selected sites, machines frequently do not work and there is no routine viral loading testing available in the country.[11] Despite the large scale up and expansion of HIV testing and subsequent increased access to ART, clinical monitoring of ART remains challenging with laboratory monitoring largely absent.

Effective ART requires optimal adherence to maintain suppression of viral replication with non-adherence resulting in treatment failure, disease progression and/or the emergence of drug resistance.[12,13] Qualitative and quantitative studies undertaken in developing countries have identified a number of common barriers to adherence including low-levels of education, low-socio-economic status, stigma, and lack of transportation to clinics, side effects, simply forgetting, and being away from home.[14,15] Some of these issues such as stigma, side effects and forgetting are also common in the developed world. Issues particularly apparent in PNG include food insecurity[16], poor road systems, long distances between villages and ART prescribing sites, delays in on-time pill pick up as a result of road blocks, tribal fighting or lack of bus fare [17], widespread use of traditional medicinal plants [18] and drug stock-out. Furthermore, a variety of Christian denominations exists in PNG with different beliefs on HIV infection and ART, which may represent additional barriers to adherence.[19]

The current paper draws from a larger study on HIV drug resistance in PNG [20] and its aims to measure ART adherence in PLHIV. We report the levels of ART adherence of PLHIV who have been taking ART using self-reporting and pill count, identified barriers to adherence, and measured the virological outcomes of PLHIV taking ART.

## Methods

### Study settings

This study was conducted in the two largest government-run sexual health and ART-administering clinics in the highlands of PNG; Goroka (Michael Alpers Clinic) in the Eastern Highlands Province and Mt Hagen (Tininga Clinic) in the Western Highlands Province. In the 2012 Global Report on HIV/AIDS, 60% of all HIV cases in PNG reported the previous year were from the highlands region, which is represented by seven provinces including those in this study. The baseline study was conducted from July 2010 to December 2011. By the end of 2011, the total number of PLHIV registered at Tininga and Michael Alpers clinics were 2,195 (67.1%, n = 1472 on ART) and 1,412 (67.3%, n = 950 on ART), respectively (extracted from clinic patient database).

### Patient recruitment and interview

Using convenience sampling, we recruited and interviewed 102 PLHIV aged ≥20 years old who were receiving ART irrespective of their time on treatment. As the study design included a follow-up component, those who mentioned possible relocation to another province in the next 12–18 months were excluded from the study. A questionnaire was administered by a trained research staff to capture participant’s demographic information, knowledge of HIV transmission, self-reported adherence, and factors influencing ART adherence, participant’s experiences with ART, and access to clinics whilst clinical information was extracted from patient charts. The questionnaire was conducted in Tok Pisin, one of three national languages in PNG. The interviews were conducted in a secluded room at the clinic sites. Ten mL of whole blood was also drawn for drug resistance and viral load testing, which were performed as described elsewhere.[20,21]

### Ethics approval

Consent was obtained from all the participants enrolled in this study. An information sheet outlining the study was read to each participant and those who agreed to participate provided a written consent and those who couldn’t write provided a thumb print. The patient consenting process was approved by the PNG Medical Research Advisory Committee (No.10.05) and the PNG Institute of Medical Research Institutional Review Board (No.1003). Processing of the blood samples in Australia was approved by the Alfred Ethics Committee (35/05) and Monash University Human Research Ethics Committee (CRF12/1712-2012000923).

### Measurement of ART adherence

ART adherence was measured using two methods: self-reported adherence captured by the questionnaire and pill counts extracted from patient medical records. In this study, self-reported adherence was defined as the patient reportedly taking all the recommended doses in the last seven days whilst self-reported time adherence was defined as taking pills on the recommended time in the last seven days. We defined “not on time” as > 1 hour from the prescribed dosage time, which was drawn from a previous study on ART in PNG. [19] At the time of recruitment, as per PNG ART treatment guidelines, patients were advised to take one pill at 7am and 7pm daily. Complete adherence was measured as not missing a dose and taking the correct dose on time in the seven days prior to participation in the study. We also measure life-time dose and time adherence as never missing a dose and never late taking medication since starting ART.

Pill count adherence is recorded routinely during monthly visits to the clinics by clinicians. Based on PNG ART treatment guidelines, patients on ART were given a month supply of pills with a buffer for two days treatment consisting of four pills (based on the treatment regimen used at that time). Adherence was measured by calculating the difference between the number of pills that were taken and the number of pills that should have been taken since the last visit (expressed as a percentage) and is categorized as poor (<90%; buffer pills + ≥ 3 doses), good (90–95%; buffer pills + 3 doses) or excellent (>95%; only buffer pills).

### Virological testing

Viral load measurements were performed on the patient plasma samples using the Exavir Load Version 3 (Cavidi AB, Uppsala, Sweden) as previously described. [21] The assay has a lower limit of detection of 200 RNA copies/mL and any reading below 200 RNA copies /mL was regarded as an undetectable viral load.

### Statistical analysis

Data were entered using Filemaker Pro 12 and uploaded and analysed in SPSS 20. Univariate analysis was performed on patient demographic information (gender, age, marital status, province of residence, religion, and employment status) and clinical parameters (time on ART, baseline CD4 T-cell count, viral load). Bivariate analysis was used to investigate associations between adherence and clinical or demographic information. The test for association was performed using Pearson chi-square. All associations were measured using a two-sided significance level of p<0.05.

## Results

### Socio-demographic characteristics of the study cohort

We recruited and interviewed 102 ART experienced PLHIV but excluded data from seven participants who had been on treatment for <3 months due to minimal time of exposure to ART. The demographic data for the remaining 95 participants is summarised in Table 1. The majority of the participants were female (63.2%, n = 60) which is reflective of the HIV epidemic in PNG.[10] Over half (52.6%, n = 50) were young people between the ages of 20–30 years of whom 78% (n = 39) were female. About fifty- two per cent (n = 49) were married and co-habiting with their spouses whilst 16.8% (n = 15) of the participants were widowed with 11 people reporting AIDS as the cause of death of their spouse. Level of highest educational attainment was low amongst the participants with 81.1% (n = 77) reporting having only had primary or no formal education. The majority (86.3%, n = 82) were not formally employed and engaged in informal small business activities or relied on their spouse, relatives or friends to support them. All the patients responded as belonging to a Christian denomination with the most common being the Seventh Day Adventist (35.8%, n = 34) followed by other international denominations (Anglican, Baptist, Catholic and Lutheran, 18.9%, n = 18) and the PNG Revival Church (13.7%, n = 13). A quarter of the participants identified with small localized churches reflecting of the diversity of Christian denominations and small outreach fellowship groups in PNG.

**Table 1:** Demographic and clinical characteristics of the study population (n = 95).

Characters	n (%)
Sex	
Female	60 (63.2)
Male	35(36.8)
Age (years)	
20–30	50 (52.6)
31–41	26 (27.4)
42–52	9 (9.5)
>52	6 (6.3)
Unknown	4 (4.2)
Marital status	
Married and living together	49 (51.6)
Widowed	16 (16.8)
Separated	13 (13.7)
Divorced	6 (6.3)
Never married	6 (6.3)
Married but not living together	5 (5.3)
Highest level of education	
No formal education	27 (28.4)
Primary school (Years 1–8)	50 (52.6)
Secondary school (Years 9–12)	16 (16.8)
Tertiary institutions*	2 (2.1)
Formal employment	
No	82 (86.3)
Yes	13 (13.7)
Religious denomination	
Seventh Day Adventist	34 (35.8)
Mainline (Anglican, Baptist, Catholic and Lutheran)	18 (18.9)
PNG Revival	13 (13.7)
Pentecostal (Foursquare and Assemblies of God)	7 (7.4)
Other international denominations	21 (22.1)
Unknown	2 (2.1)
Time on ART(months)	
3–12	27 (28.4)
13–24	26 (27.4)
25–36	26 (27.4)
>37	16 (16.8)
Baseline CD4 T-cell count^**^(cells/μL)	
<100	11 (11.6)
100–300	28 (29.5)
301–501	20 (21.0)
>501	8 (8.4)
Unknown	28 (29.5)
Viral load (RNA copies/mL)	
<200 (undetectable)	78 (82.1)
≥ 200 (detectable)	17 (17.9)
Unknown	1 (1.1)
Failing therapy***	
No	82 (86.3)
Yes	13 (13.7)

*Tertiary institutions includes Technical/Vocational and University.

**Baseline CD4 T-cell count prior to ART initiation.

***Patients on ART ≥6 months on ART and having >1000 RNA copies/mL.

### Participants ART experience, history and access

Information on ART experience and knowledge, treatment history and access to treatment was gathered to capture the experiences of patients on ART and how this may affect long-term treatment adherence. There were 88 participants who responded to the question on whether ART had improved their health with most 95.5% (n = 84) noting that ART had greatly improved their health. Of those participants whose treatment regimen history was recorded (n = 93), the majority (82.8%, n = 77) had remained on first line therapy since ART initiation. Of those who had not (17.2%, n = 16), 10 had reasons recorded for treatment change, of which most were due to drug stock outs, and non-specific drug side effects. Of the different health issues reported, most participants (81.1%, n = 77) reported memory loss, tingling in hands and feet, nausea, bloated stomach and headaches. Thirty seven percent (n = 35) of the participants sought Christian faith healing, five (5.3%) consulted traditional spiritual healers and six (6.3%) took traditional herbs and juices as alternate HIV treatment at some point while on ART. At the time of recruitment, 11.7% (n = 11) of the participants were concurrently being treated for co-infections such as tuberculosis, pneumonia, malaria and typhoid. In addition, three (4.8%) participants were taking treatment for sexually transmitted infections. The majority (87.1%, n = 54) commenced cotrimoxazole as prophylaxis for bacterial infections as recommended by WHO. Thirty seven per cent (n = 34) of participants reported facing difficulty getting to the clinics. Very few (28.3%, n = 26) mentioned belonging to a support group whilst 57.6% (n = 53) of the patients had never heard of drug resistance and didn’t know what it was. In summary, whilst most of the participants reported improved health after starting ART, others experience side-effects common to first line drugs and sought other forms of treatment/healing such as herbal therapies and prayer.

### Immunological and virological characteristics of the participants

Pre-ART initiation (baseline) CD4 T-cell count was available for 67 participants who had been on therapy for a median of 20 months (range; 3–36). The median CD4 T-cell count for these participants at the time of ART-initiation was 264 cells/mm^3^ (range; 15–836) with 11 (16.4%) having a CD4 T-cell count of <100 cells/mm^3^. Eighty two per cent (n = 78/95) had undetectable viral load (<200 copies RNA/mL) at the time of recruitment and, based on the WHO definition of virological failure (on ART for ≥ 6 months and having ≥1000 copies RNA/mL), 13.7% (n = 13) of participants were failing therapy.

### Participants knowledge of HIV transmission

We asked questions regarding HIV transmission and acquisition to understand the level of HIV related knowledge in our study population. The study participants were asked to volunteer answers without prompting. Correct knowledge was not particularly high; the areas where people displayed the greatest knowledge were in relation to condoms preventing transmission (63.2%, n = 60), limiting sex to one faithful uninfected partner (40.0%, n = 38) and abstaining from sex (28.4%, n = 27). Ten (10.5%) participants stated going to church as a way people can avoid getting HIV. Table 2 summarizes participants’ HIV transmission-related knowledge. Furthermore, participants were asked whether a pregnant mother living with HIV can transmit HIV to her unborn child and of the 92 participants who responded to the question, 65.2% (n = 60) gave the correct answer, of which a greater proportion (63.3%, n = 38) were female. The proportion of women is higher than men in this study, and female participants may have attended antenatal clinics and heard HIV transmission prevention messages, therefore affecting their responses to the questions.

**Table 2:** Participants’ responses to ways of avoiding HIV transmission (n = 95).

Responses*	n (%)
Condoms	60 (63.2)
Limiting sex to one faithful uninfected partner	38 (40.0)
Abstinence from sex	27 (28.4)
Avoid sharing razor blades/needles/tooth brush	20 (24.4)
Limiting number of sexual partners	12 (14.6)
Avoid touching fresh cuts/blood	11 (13.4)
Go to church	10 (12.2)
Avoiding sex with prostitutes	9 (11.0)
Changing sexual behaviours	9 (11.0)
Avoid blood transfusion	2 (2.4)
Don’t get married or have boyfriend or girlfriend	2 (2.4)
Avoid sharing clothes, beddings, and eating utensils	2 (2.4)
Other	12 (14.6)

*Participants were allowed to provide multiple responses

### Levels of ART adherence

Of the 91 participants who responded to self-reported adherence questions, 82.4% (n = 75) reported never missing a dose in the last seven days and 67.0% (n = 61) stated they had never been late at taking their medication. Perfect adherence (defined as taking all doses on time in the last seven days) was reported by 56.0% (n = 51) participants. Eighty five of the 95 participants had records of pill counts in the last month, of which, 40.0% (n = 34) had excellent adherence (>95%; patients returning to the clinic after a month with the correct number of pills). There was poor agreement between self-report and pill count measures of adherence with only 33.3% (27/81) of participants having excellent adherence defined by both measures with no significant association between self-reported adherences and pill count detected.

We tested whether any demographic or behavioural factors were associated with complete adherence and found taking prescribed medications apart from ART was significantly associated with self-reported adherence (χ^2^ 6.166; p = 0.01, S1 Table) whilst patients who had been on ART for ≥ 1 year were more likely to be adherent by pill count (χ^2^ 6.081; p = 0.014, S2 Table). There were no significant associations with any other socio-demographic or clinical parameters and adherence.

### Correlates of virological failure

Given 13.7% of participants exhibited virological failure, we investigated what factors might correlate with this and the results are summarized in S3 Table. Participants who had reached higher education levels (secondary or tertiary colleges; χ^2^7.4003; p = 0.021) and participants who held formal employment (χ^2^7.828; p = 0.05) were more likely to have virological failure. Participants who had heard of drug resistance (χ^2^4.521; p = 0.033) and those who had remained on the same treatment since starting ART (χ^2^12.797; p = 0.001) were less likely to experience virological failure. No other socio-demographic factors were significantly associated with virological failure (S3 Table).

### Barriers and facilitators to ART adherence

In this study, participants were asked to cite barriers or facilitators to treatment adherence relating to their life-time adherence to ART. They were asked to state the reasons for any missed doses since starting treatment. Of the 50 participants who stated having missed doses, 47 identified factors that affected their adherence (summarised in Table 3). The most common responses mentioned were “being forgetful” (42.6%, n = 20), “busy with work” (27.7%, n = 13), “leaving medications at home” (17.0%, n = 8), “no medication left” (17.0%, n = 8) and “medicine-related” (17.0%, n = 8).

**Table 3:** Barriers to life time adherence to ART (n = 47).

Reasons for missing doses*	n (%)
I forgot	20 (42.6)
I was busy with work	13 (27.7)
I left medicines at home	8 (17.0)
No medication left/ not available	8 (17.0)
Medicine-related#	8 (17.0)
Not enough food /no water available	7 (14.9)
I believed God has healed me/prayer/fasting	5 (10.6)
I was busy with children/grandchildren	4 (8.5)
I was drunk/stoned	4 (8.5)
I was very sick	3 (6.4)
People were around	2 (4.3)
Other	7 (14.9)

* Participants were allowed to cite more than one reason for missing doses.

# Includes, I vomit when I take medicine, I don’t feel well with medicine, medicines are hard to swallow, medicine reminds me of my HIV status and I was taking medicines for other illness.

We also recorded self-reported time adherence from each individual. Fifty two (55.9%) patients admitted never taking ART on time since initiating ART. The common reasons cited were; “I forgot” (32.7%, n = 16), “busy with work” (26.5%, n = 13) and “not having a watch”/watch related issues (22.4%, n = 11).

Forty-one participants (43.2%) reported never missing doses since ART initiation and the most common reasons were; “I don’t want to die” (85.4%, n = 35), “I wanted to follow doctor’s advice” (80.5%, n = 33), “I wanted to look healthy” (65.9%, n = 27), and 56.1% (n = 23) cited “wanted to stay alive to see my children grow” and “I wanted medicine to work effectively”. These study participants who never missed ART doses also cited that they get treatment support from health workers (51.2%, n = 21), families (41.5%, n = 17) and church groups (4.1%, n = 14) which assisted them with maintaining adherence. Other reasons why patients never missed their doses of ART is summarised in Table 4.

**Table 4:** Facilitators of life time adherence to ART (n = 41).

Reasons to never missing doses*	n (%)
I don’t want to die	35 (85.4)
I wanted to follow doctor’s advice	33 (80.5)
I wanted to look healthy	27 (66.0)
I wanted to stay alive to see my children grow	23 (56.1)
I wanted medicine to work effectively	23 (56.1)
I wanted to continue to work	22 (54.0)
I get support from the health workers	21 (51.2)
I get support from my family	17 (41.5)
I get support from the church	14 (31.4)
I get support from the community	11 (27.0)
Other	11 (27.0)

* Participants were allowed to cite more than one reason for never missing doses.

## Discussion

The purpose of this study was to measure the levels of adherence to ART in PLHIV in the highlands of PNG using self-reporting and pill count. In addition, the study sought to identify factors that hinder patients’ ability to adhere to treatment and to determine their virological outcomes. According to self-reported adherence data, the study cohort seemed to be fairly adherent, with 78.9% of participants self-reporting full dose adherence in the last seven days. Consistent with this findings, Kelly *et al* (2010) in the first ART adherence study in PNG found 85.0% of study subjects from the highlands of PNG self-reported as being adherent in the past seven days. [19] Also consistent with self-reported adherence levels from our study, previous studies conducted in resource limited settings in Africa also reported have self-reported dose adherence levels between 81–96%. [22–25]

Whilst self-reported ART adherence levels in this study were similar to that previously reported for PNG and also to studies undertaken in Africa, this study has highlighted significant discrepancies between self-reported and pill-count adherence (discussed further below). In the present study, the number of participants who self-reported adherence was higher compared to the pill count measure of adherence. Although self-reporting is the most frequently used measure of adherence because it is inexpensive and easy to administer, it is subjective and is prone to bias, often resulting in overestimation. [26] Meanwhile, hospital pill count is measured by clinicians and may be more accurate, however, it can be masked by “pill dumping”.[26] We acknowledge that the different length of time measure for each method (seven days for self-report and one month for pill count) to assess adherence in this study could possibly have contributed to the difference in adherence rate, as there is obviously a greater risk of missing dose over a month. As it is, adherence is often difficult to measure and thus requires combined multiple approaches to measure adherence including qualitative measures. As a vital component of HIV/AIDS care and treatment in PNG, ART adherence assessments need to be prioritised and strengthened. Pill count seemed to be the only method of adherence assessment in ART clinics in PNG and should be an active part of treatment care, incorporating extra adherence measures such as patient diaries and self-report into routine patient clinic visit.

Reasons for missing doses observed in PNG were similar to those previously identified from both developed and developing countries with over half of the participants in this study who reported missing ART doses citing forgetfulness and being busy at work as major factors affecting ability to adhere. [22,25,27] The earlier study on ART adherence in PNG found the most common reasons for missing doses were forgetfulness and not having available food. The same study also reported that participants in the National Capital District (in the southern region of PNG) were more likely to experience problems with food insecurity compared to the other regions.[16] The present study was conducted in the Highlands Region of PNG and food insecurity was cited by less than ten percent of the participants. This is likely because many Highlanders have gardens and food is more abundant. A review by Mills *et al* reported similar barriers in both the developed and developing world; however, long travel distance and financial constraints were unique barriers cited in resource poor settings. [28] At the time of recruitment, 37% of our participants mentioned experiencing difficulty with transportation to access treatment and not having bus fares or having to travel long distances, which are also barriers consistently reported in developing countries.[28] The PNG National Department of Health, recognizing this is a major barrier, has since decentralized HIV/AIDS treatment and care to the district levels in some provinces so that participants have less distance to travel. However, travelling to clinics is still likely to present a barrier to adherence due to the particularly rugged terrain in PNG where villages are isolated from each other by mountainous ranges, poor road systems and transportation costs are generally high in PNG. This barrier will need continual monitoring as treatment programs continue to expand.

It has been reported that not only is pill taking important but that adherence to dose timing is crucial for obtaining undetectable viral load, [29] hence why it was included in the first ART study in PNG and subsequently in this one. [19] This could present a problem in the PNG context, as evident by the fact that in our study more than half of the participants reported never taking ART medications on time. Apart from patients being forgetful and busy with work, almost a quarter of participants reported not having a watch or clock available and reported to guessing the correct to take their medication. Participants living in rural areas cited relying on the sunrise and sunset and school, church and community hall bells to remind them to take their medication, however, these timer reminders are not reliable and may interfere with adherence. An approach that could be explored to improve time adherence and subsequent viral suppression is the use of mobile phone text message reminders which have been reported to result in improved ART adherence in patients in other similar settings.[27,30]

In countries with strong religious beliefs, religion can influence a patient’s behaviour towards their medication. [31] This association was evident in an earlier study of ART adherence in PNG, which found that people attending the Revival church were more likely to miss their dose.[19] Although all of the participants in the current study were affiliated with a Christian denomination, there was no association between any particular denomination and adherence; however, high diversity and small sample size made this difficult to assess statistically. Misconceptions were still evident with three people citing they believed God had healed them of HIV, and had subsequently discontinued treatment for a period of time and later resumed their medication based on their clinical history notes. The Revival church in PNG emphasises faith healing and discourages patients from taking their ART. [32] The number of participants belonging to this church was small (n = 13) in this study, making associations between this religion and ART adherence/treatment success difficult to investigate. Both negative and positive associations between adherence and religion are evident in other settings. Studies in Africa have found that people became more religious after they were diagnosed with HIV and consequently sustained good adherence[31] whilst a study in Kampala reported some patients discontinued ART because they claimed to be cured from HIV after prayers.[33,34] About 10 per cent of the participants in our study mentioned ‘going to church’ as a way to avoid getting HIV whilst 31% mentioned that they get support for adhering to ART from church. Churches have great potential to play either positive or negative role in HIV treatment programs in PNG [35] and should be harnessed by programs to focus on positive support for PLHIV such as community support, encouragement, and social change.

Most of the patients who never missed their doses in this study were driven by personal positive thoughts towards well-being. Between 27–51% of patients mentioned that getting support from health workers, family and church helped them to adhere to their treatment. Studies have shown that self-perceived wellness and family support are associated with treatment adherence [24] whilst a lack of social support has been associated with non-adherence. [36] A study in South Africa among 268 patients showed improved ART adherence outcomes with better support from the community, community health worker, treatment buddy, and support group.[37] Very few patients (23.4%) in our study mentioned belonging to a formal support group, highlighting an important opportunity for future strategies to improve ART adherence in PNG.

The majority of patients in our study were still on first line therapy after a median of 20 months on ART with 83% having an undetectable viral load; however, 14% had virological failure. A similar study performed in Dar-es-Salaam, Tanzania, reported 15% virological failure rates amongst patients who have been on ART for a median of 20 months.[38] Our study found that participants who had higher levels of education or held formal employment were more likely to experience virological failure; however, we acknowledge that the number of participants who had secondary school and tertiary education or formal employment was small (n = 13). Conversely, participants who had heard of drug resistance and those whose treatment regimen has been constant since initiation ART were significantly less likely to have virological failure. These findings suggest a greater awareness of the potential for drug resistance may improve adherence and thus treatment outcomes. Taken together, these data are encouraging in that they indicate that the majority of PLHIV in our study cohort maintained virologically suppression on their initial ART regimen, indicating that first-line treatment in PNG is still largely effective. However, vigilance must remain as there is already evidence of drug resistance within the same study cohort.[20]

We acknowledge that our study population was small and thus we could not perform multivariate analysis, therefore, our data should be interpreted with caution. A larger cohort of patients with a study questionnaire incorporating standardized and widely used instrument for measuring self-report adherence such as that produced by the Adult AIDS Clinical Trial Group [39] may identify factors in the future that correlates with adherence and clinical outcomes in PNG and provide consistency with other global studies measuring treatment adherence.

In conclusion, a high level of self-reported adherence was apparent amongst our study respondents amidst the socio-economic challenges, and cultural and religious complexities that exist in the country, although adherence measures by pill counts suggests more work needs to be done to achieve maximum adherence. Whilst the majority of study participant were knowledgeable about HIV transmission, there is a need for continued education and counselling on this topic amongst PLHIV since many still hold misconceptions about how to avoid HIV infection. In-depth education on the consequences of non-adherence remains important for patients initiating treatment. Self-motivated positive thoughts and support from family, health care workers, churches, and community can play a significant role and could be further harnessed to improve treatment outcomes. Advocacy and involvement of the above-mentioned groups needed to be emphasised in the national HIV care and treatment policies in PNG to help maintain ART adherence and thus efficacy.

## Supporting Information

S1 Table Correlates of patient self-reported ART adherence in the last week with demographic and clinical characteristics (n = 91). (DOCX)

S2 Table Correlates of pill count adherence with patient demographic and clinical characteristics (n = 85). (DOCX)

S3 Table Correlates of virological failure with demographic and clinical characteristics (n = 95). (DOCX)
