# Supplementary material for: Factors Influencing Antiretroviral Adherence and Virological Outcomes in People Living with HIV in the Highlands of Papua New Guinea
Source: PLoS One. 2015 Aug 5;10(8):e0134918. doi: 10.1371/journal.pone.0134918 (PMC4526685; doi:10.1371/journal.pone.0134918)
Supplement: S1 Table — (DOCX) [file pone.0134918.s001.docx]

**S1 Table. Correlates of patient self-reported ART adherence in the last week with demographic and clinical characteristics (n=91).**

| Characters | No. of patients (%)  Adherent * Non-adherent | | Test of significance at p<0.05  Pearson Chi square (χ^2^) |
| --- | --- | --- | --- |
| Sex |  |  | χ^2^ 0.427;df 1; p=0.514 |
| Female | 45 (80.0) | 11 (19.6) |  |
| Male | 30 (85.7) | 5 (14.3) |  |
|  |  |  |  |
| Age group, n=88 |  |  | χ^2^1.128; df 1; p=0.288 |
| <30 years | 30 (76.9) | 9 (23.1) |  |
| ≥ 30 years | 42 (85.7) | 7 (14.3) |  |
|  |  |  |  |
| Education |  |  | χ^2^ 2.430; df 2; p=0.297 |
| No formal education | 19 (79.2) | 5 20.8) |  |
| Primary school (1-8) | 43 (87.8) | 6 (12.2) |  |
| Secondary school (9-12) + above | 13 (72.2) | 5 (27.8) |  |
|  |  |  |  |
| Formal employment |  |  | χ^2^ 0.51df 1; p=0.822 |
| No | 64 (82.1) | 14 (17.9) |  |
| Yes | 11 (84.6) | 2 (15.4) |  |
|  |  |  |  |
| Province of residence |  |  | χ^2^4.017; df 2; p=0.134 |
| Eastern Highlands | 37 (88.1) | 5 (11.9) |  |
| Western Highlands | 36 (80.0) | 9 (20.0) |  |
| Others | 2 (50.0) | 2 (50.0) |  |
|  |  |  |  |
| Time on ART |  |  | χ^2^1.238; df 4; p=0.266 |
| <1 year | 18 (75.0) | 6 (25.0) |  |
| ≥ 1year | 57 (85.1)) | 10 (14.9) |  |
|  |  |  |  |
| Baseline CD4 T cell |  |  | χ^2^0.002; df 1; p=0.968 |
| count(cells/mm^3^),n=67 |  |  |  |
| <200 | 22 (81.5) | 5 (18.5) |  |
| ≥200 | 30 (81.1) | 7 (18.9) |  |
| Viral load (RNA copies/mL), n=90 |  |  | χ^2^0.0973; df 1; p=0.324 |
| <200 (Undetectable) | 63 (85.1) | 11(14.9) |  |
| ≥200 (Detectable) | 12 (75.0) | 4 (25.0) |  |
|  |  |  |  |
| Virological failure |  |  | χ^2^1.820; df 1; p=0.177 |
| No | 66 (84.6) | 12 (15.4) |  |
| Yes | 9 (69.2) | 4 (30.8) |  |
|  |  |  |  |
| Pill count, n=81 |  |  | χ^2^0.007; df 1; p=0.933 |
| Adherent | 27 (84.4) | 5 (15.6) |  |
| Non-adherent | 41 (83.7) | 8 (16.3) |  |
|  |  |  |  |
| ART side effects, n=87 |  |  | χ^2^ 0.045; df 1; p=0.832 |
| Present | 56 (81.2) | 13 (18.8) |  |
| Absent | 15 (83.3) | 3 (16.7) |  |
|  |  |  |  |
| Treatment change, n=89 |  |  | χ^2^ 0.002; df 1; p=0.969 |
| No | 59 (81.9) | 13 (18.1) |  |
| Yes | 14 (82.4) | 3 (17.6) |  |
|  |  |  |  |
| Taking other medications apart from ART, n=90 |  |  | χ^2^6.166; df 1; p=0.013 |
| Yes | 52 (89.7) | 6 (10.3) |  |
| No | 22 (68.8) | 10 (31.2) |  |
|  |  |  |  |
| Sought other forms of therapy/healing |  |  | χ^2^ 0.427; df 1; p=0.514 |
| Yes | 30 (85.7) | 5 (14.3) |  |
| No | 45 (80.4) | 11 (19.6) |  |
|  |  |  |  |
| Difficulty getting to the clinic |  |  | χ^2^ 0.471; df 1; p=0.493 |
| No | 49 (84.5) | 9 (21.2) |  |
| Yes | 26 (78.8) | 7 (21.2) |  |
|  |  |  |  |
| Member of a support group |  |  | χ^2^0.741; df 1; p=0.389 |
| No | 53 (80.3) | 13 (19.7) |  |
| Yes | 22 (88.0) | 3 (19.7) |  |
|  |  |  |  |
| Ever heard of drug resistance |  |  | χ^2^2.242; df 1; p=0.132 |
| No | 41 (62.377.4) | 12 (22.6) |  |
| Yes | 34 (89.5) | 4 (10.5) |  |

*Self-reported adherence in this study is measured as not missing doses in the past 7 days.
