# Supplementary material for: Factors Influencing Antiretroviral Adherence and Virological Outcomes in People Living with HIV in the Highlands of Papua New Guinea
Source: PLoS One. 2015 Aug 5;10(8):e0134918. doi: 10.1371/journal.pone.0134918 (PMC4526685; doi:10.1371/journal.pone.0134918)
Supplement: S2 Table — (DOCX) [file pone.0134918.s002.docx]

**S2 Table. Correlates of pill count adherence with patient demographic and clinical characteristics (n=85).**

| Characters | No. of patients (%)  Adherent * Non-adherent | | Test of significance at p<0.05  Pearson Chi square (χ^2^) |
| --- | --- | --- | --- |
| Sex |  |  | χ^2^ 0.000;df 1; p=1.000 |
| Female | 22 (40.0) | 33 (60.0) |  |
| Male | 12 (40.0) | 18 (60.0) |  |
|  |  |  |  |
| Age group, n=80 |  |  | χ^2^ 0.005; df 1; p=0.941 |
| <30 years | 14 (37.8) | 23 (62.2) |  |
| ≥30 years | 17 (38.6) | 27 (61.4) |  |
|  |  |  |  |
| Education |  |  | χ^2^ 1.050; df 2; p=0.591 |
| No formal education | 9 (39.1) | 14 (60.9) |  |
| Primary school (1-8) | 21 (43.8) | 27 (56.2) |  |
| Secondary school (9-12) and above | 4 (28.6) | 81 (71.4) |  |
|  |  |  |  |
| Formal employment |  |  | χ^2^ 0.157; df 1; p=0.692 |
| No | 29 (39.2) | 45 (60.8) |  |
| Yes | 5 (45.5) | 6 (54.5) |  |
|  |  |  |  |
| Province of residence |  |  | χ^2^ 0.449; df 1; p=0.799 |
| Eastern Highlands | 16 (42.1) | 22 (57.9) |  |
| Western Highlands | 17 (39.5) | 26 (60.5) |  |
| Others | 1 (25.0) | 3 (75.0) |  |
|  |  |  |  |
| Time on ART |  |  | χ^2^ 4.358; df 1; p=0.037 |
| < 1 year | 4 (20.0) | 16 (80.0) |  |
| ≥ 1 year | 30 (46.2) | 35 (53.8) |  |
|  |  |  |  |
| Baseline CD4 T cell count, n=60 |  |  | χ^2^0.533; df 1; p=0.465 |
| <200 | 9 (40.9) | 13 (59.1) |  |
| ≥200 | 12 (31.6) | 26 (68.4) |  |
|  |  |  |  |
| Viral load (RNA copies/mL), n=84 |  |  | χ^2^ 0.158; df 1; p=0.691 |
| <200 (Undetectable) | 29 (41.4) | 41 (58.6) |  |
| ≥200 (Detectable) | 5 (35.7) | 9 (64.3) |  |
|  |  |  |  |
| Ever missed a dose in the last week, n=81 |  |  | χ^2^ 0.007; df 1; p=0.933 |
|  |  |  |  |
| No (adherent) | 27 (39.7) | 41(60.3) |  |
| Yes (non-adherent) | 5 (38.5) | 8 (61.5) |  |
|  |  |  |  |
| Virological failure |  |  | χ^2^ 0.70; df 1; p=0.792 |
| No | 30 (40.5) | 44 (59.5) |  |
| Yes | 4 (36.4) | 7 (63.6) |  |
|  |  |  |  |
| ART side effects, n=81 |  |  | χ^2^ 0.075;df 1; p=0.784 |
| Present | 26 (40.0) | 39 (60.0) |  |
| Absent | 7 (43.8) | 9 (56.2) |  |
|  |  |  |  |
| Treatment change |  |  | χ^2^ 0.115; df 1; p=0.734 |
| Yes | 7 (43.8) | 9 (56.2) |  |
| No | 27 (39.1) | 42 (60.9) |  |
|  |  |  |  |
| Taking other medications apart from ART, n=84 |  |  | χ^2^ 1.118; df 1; p=0.290 |
| Yes | 20 (36.4) | 35 (63.6) |  |
| No | 14 (48.3) | 15 (51.7) |  |
|  |  |  |  |
| Sought other forms of therapy/healing |  |  | χ^2^ 0.287; df 1; p=0.592 |
| Yes | 16 (43.2) | 21 (56.8) |  |
| No | 18 (37.5) | 30 (62.5) |  |
|  |  |  |  |
| Difficulty getting to the clinic, n=82 |  |  | χ^2^ 0.111; df 1; p=0.740 |
| No | 21 (40.4) | 31 (59.6) |  |
| Yes | 11 (36.7) | 19 (63.3) |  |
|  |  |  |  |
| Member of a support group, n=82 |  |  | χ^2^ 0.089; df 1; p=0.765 |
| No | 24 (40.0) | 36 (60.0) |  |
| Yes | 8 (36.4) | 14 (63.6) |  |
|  |  |  |  |
| Ever heard of drug resistance, n=81 |  |  | χ^2^ 0.024; df 1; p=0.876 |
| No | 18 (38.3) | 29 (61.7) |  |
| Yes | 14 (40.0) | 21 (60.0) |  |

* ≥95 % (adherence) and <95% pills (non-adherence) in the last month.
