# Supplementary material for: Factors Influencing Antiretroviral Adherence and Virological Outcomes in People Living with HIV in the Highlands of Papua New Guinea
Source: PLoS One. 2015 Aug 5;10(8):e0134918. doi: 10.1371/journal.pone.0134918 (PMC4526685; doi:10.1371/journal.pone.0134918)
Supplement: S3 Table — (DOCX) [file pone.0134918.s003.docx]

**S3 Table. Correlates of virological failure with demographic and clinical characteristics (n=95)**

| Characters | No. of patients (%)  Yes No | | Test of significance at p<0.05  Pearson Chi square (χ^2^) |
| --- | --- | --- | --- |
|  |  | |  |
| Sex |  |  | χ^2^ 1.871;df 1; p=0.171 |
| Female | 6 (10.0) | 45 (90.0) |  |
| Male | 7 (20.0) | 28 (80.0) |  |
|  |  |  |  |
| Age group, n=91 |  |  | χ^2^ 0.266; df 1; p=0.606 |
| <30 years | 5 (12.2) | 36 (87.8) |  |
| ≥30 years | 8 (16.0) | 42 (84.0) |  |
|  |  |  |  |
| Education |  |  | χ^2^ 7.403; df 2; p=0.025 |
| No formal education | 3 (11.1) | 24 (88.9) |  |
| Primary school (1-8) | 4 (8.0) | 46 (92.0) |  |
| Secondary school (9-12) and above | 6 (33.3) | 12 (66.7 ) |  |
|  |  |  |  |
| Formal employment |  |  | χ^2^ 7.828; df 1; p=0.005 |
| No | 8 (9.8) | 74 (90.2) |  |
| Yes | 5 (38.5) | 8 (61.5) |  |
|  |  |  |  |
| Province of residence |  |  | χ^2^ 1.150; df 2; p=0.563 |
| Eastern Highlands | 8 (16.7) | 40 (83.3) |  |
| Western Highlands | 5 (11.6) | 38 (88.4) |  |
| Others | 0 (0.0) | 4 (100.0) |  |
|  |  |  |  |
| Time on ART |  |  | χ^2^ 0.778; df 1; p=0.378 |
| <1 year | 2 (8.3) | 22 (91.7) |  |
| ≥1 year(s) | 11 (15.5) | 60 (84.5) |  |
|  |  |  |  |
| Baseline CD4 T cell count, n=67 |  |  | χ^2^0.159; df1; p=0.690 |
| <200 | 4 (14.3) | 24 (85.7) |  |
| ≥200 | 7 (17.9) | 32 (82.2) |  |
| Viral load (RNA copies/mL), n=92 |  |  | χ^2^ 60.543 df 1; p=0.001 |
| <200 (Undetectable) | 1 (1.3) | 77 (98.7) |  |
| ≥200 (Detectable) | 12 (75.0) | 4 (25.0) |  |
|  |  |  |  |
| ART adherence in the last week, n=91 |  |  | χ^2^1.820; df 1; p=0.177 |
| Adherent | 9 (12.0) | 66 (88.0) |  |
| Non-adherent | 4 (25.0) | 12 (75.0) |  |
|  |  |  |  |
| Pill count, n=85 |  |  | χ^2^1.070; df 1; p=0.792 |
| Adherent | 4 (11.8) | 30 (88.2) |  |
| Non-adherent | 7 (13.7) | 44 (86.3) |  |
|  |  |  |  |
| ART side effects, n=93 |  |  | χ^2^ 0.185; df 1; p=0.667 |
| Present | 11 (15.1) | 62 (84.9) |  |
| Absent | 2 (11.1) | 16 (88.9) |  |
|  |  |  |  |
| Treatment change, n=93 |  |  | χ^2^ 12.797; df 1; p=0.001 |
| Yes | 7 (41.2) | 10 (58.2) |  |
| No | 6 (7.9) | 70 (92.1) |  |
|  |  |  |  |
| Taking other medications apart from ART, |  |  | χ^2^ 1.339; df 1; p=0.247 |
| n=94 |  |  |  |
| Yes | 6 (9.8) | 55 (90.2) |  |
| No | 6 (18.2) | 27 (81.1) |  |
|  |  |  |  |
| Sought other forms of therapy/healing, |  |  | χ^2^ 0.001; df 1; p=0.969 |
| n=95 |  |  |  |
| Yes | 5 (13.5) | 32 (86.5) |  |
| No | 8 (13.8) | 50 (86.2) |  |
|  |  |  |  |
| Difficulty getting to the clinic, n=92 |  |  | χ^2^ 0.412; df 1; p=0.082 |
| No | 11 (19.0) | 47 (81.0) |  |
| Yes | 2 (5.9) | 32 (94.1) |  |
|  |  |  |  |
| Member of a support group, n=92 |  |  | χ^2^ 0.201; df 1; p=0.654 |
| Yes | 3 (11.5) | 23 (88.5) |  |
| No | 10 (15.2) | 56 (88.4) |  |
|  |  |  |  |
| Ever heard of drug resistance, n=92 |  |  | χ^2^ 4.521; df 1; p=0.033 |
| Yes | 2 (5.1) | 37 (94.9) |  |
| No | 11 (20.8) | 42 (79.2) |  |

*Virological failure is defined as on ART for ≥6months and having ≥1000 RNA copies/mL.
